# A method for assessing chemically-induced paralysis in headless mosquito larvae

**DOI:** 10.1016/j.mex.2014.12.002

**Published:** 2014-12-12

**Authors:** Rafique M. Islam, Jeffrey R. Bloomquist

**Affiliations:** Neurotoxicology Laboratory, Department of Entomology and Nematology, Emerging Pathogens Institute, University of Florida, Gainesville, FL 32611, United States

**Keywords:** Insect bioassay, Toxicology, Pharmacology

## Abstract

There is a growing interest in studies of mosquito physiology and toxicology due to the heightened need for controlling this group of human disease vectors. In the process of testing a group of polar compounds on mosquito muscles, a novel headless larva bioassay was developed. The heads were removed from fourth instar *Aedes aegypti* larvae, which permitted access of pharmacological agents to the hemocoel while maintaining larval viability. The method allowed effective quantification of the paralytic actions of water soluble compounds that could not ordinarily penetrate the mosquito larva integument and was more easily performed than injection when studying small, soft-bodied aquatic organisms.

The summary of the method is:

• Heads of *A. aegypti* larvae were detached with two pairs of forceps, and the larvae remained responsive for at least 5 h.
• The responsiveness of the larvae was assessed by using a microscope to observe movement after the larvae were probed with an insect pin.
• Drug effects were quantified using either a binary paralysis determination (paralyzed *vs*. not paralyzed), or by counting movement units after probing.

## Graphical abstract

**Figure:**
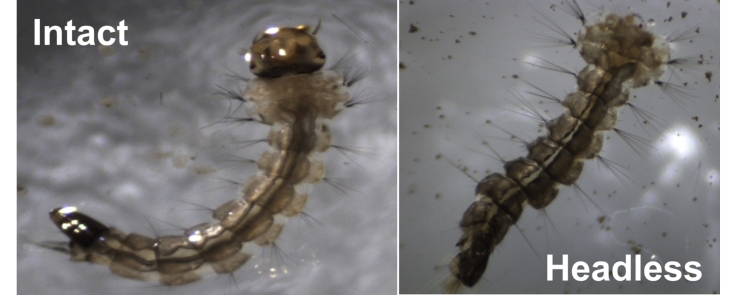

## Method details

### Introduction

Initial efforts to study the toxicity of water soluble compounds in intact mosquito larvae were not successful due to poor penetration of the test compounds through the larval cuticle. Since the cuticle is known to be a major barrier for chemical penetration in insects [1] and nematodes [2], most investigators bypass this barrier by the use of an injection bioassay, a common protocol used to evaluate the effects of venoms [3]. To overcome the weak response of nicotinic acetylcholine receptor ligands in *Caenorhabditis elegans*, Lewis et al. [4] introduced a cut-worm model, which was further developed by Ruiz-Lancheros et al. [5]. According to the refined method, *C. elegans* were severed at approximately one-third of their length between the anterior end and mid-point, and the method improved response time and reduced the amount of drug used [5]. The hypothesis that detaching the heads of mosquito larvae would provide a diffusion pathway into the hemocoel to facilitate the analysis of toxic chemical properties was tested in the present study.

## Preparation of *Aedes aegypti* larvae

Early stage (1st or 2nd instar) *A. aegypti* larvae were obtained from the United States Department of Agriculture, Agricultural Research Service (USDA ARS, Gainesville, FL, USA). Larvae were harvested in unfiltered tap water and held in large trays at approximately 25 °C, and supplemented with food consisting of 3 parts liver powder (MP Biomedical, Solon, OH, USA) and 2 parts Brewer’s yeast (MP Biomedical). For all experiments, fourth instar larvae were used. Removal of heads was performed by pressing the neck of the larva against a Sylgard (RTV 615A/B, General Electric Corp., Wafford, NY) surface in a 35 mm dish (Becton Dickinson Labware, Franklin Lakes, NJ, USA) with one pair of forceps and pulling the head away with another pair. Because larvae are actively in motion in water, we reduced this movement by keeping the larvae in a minimal amount of liquid while removing the heads. Headless larvae (*n* = 10) were transferred to a small glass chamber containing 1 ml of a mosquito larval physiological saline solution containing NaCl = 0.9%, CaCl_2_ = 0.02%, KCl = 0.02%, NaHCO_3_ = 0.01%, pH 6.9 [6]. The saline was tested each time for correct pH before use. All saline components were purchased from commercial suppliers.

## Validation of the assay: larval survival time and behavioral responses

**Fig. 1:**
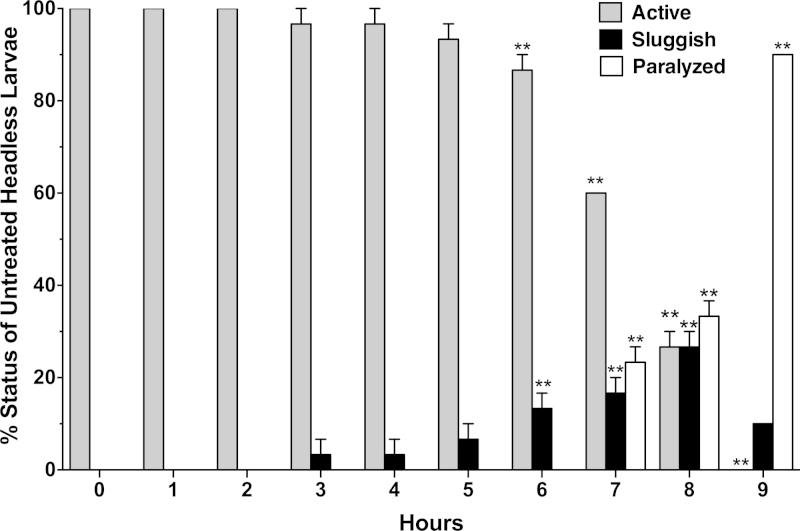
Condition of 4th instar headless *Aedes aegypti* larvae in saline without any chemical treatment over a 9 h period. Batches of 10 larvae were analyzed for responses to probing and percentage responses of 3 groups were averaged. The means (*n* = 3 batches of 10 larvae each) of “active”, “sluggish”, and “paralyzed” larvae are presented at hourly intervals (with SEM). A repeated measures ANOVA with Dunnett’s posttest comparing all means to 0 h controls was performed using Prizm™ software (GraphPad Software, San Diego, CA, USA). Asterisks (**P < 0.01) for active larvae indicate time points where the proportion active was significantly less than controls. For sluggish and paralyzed categories, asterisks indicate a significant increase in that condition (**P < 0.01) compared to controls.

A standard dissecting microscope (World Precision Instruments, Inc., Sarasota, FL, Model PZMT111) and video camera with computer interface (Model DFK 31AU03, The Imaging Source, Bremen, Germany, World Precision Instruments, Inc., Sarasota, FL) was used to observe the movement of the larvae and document behavior. While an intact mosquito larva was highly active and showed rapid swimming motion in water (Video 1), an untreated headless larva tended to be less spontaneously mobile, but responded vigorously when probed with an insect pin (Video 1). The response to probing of headless larvae (Video 1) fell into one of three categories (Fig. 1). A non-paralyzed larva initiated rapid bilateral contractions of the abdominal segments (“active”). The second category was “paralyzed” (or dead) if the larvae did not move after probing with an insect pin. Finally, it was observed that some headless larvae displayed an attenuated response to probing, and were considered “sluggish” but not paralyzed. These sluggish larvae, along with the active larvae, were considered unaffected for the purpose of drug effect quantitation. Over 90% of the untreated, headless larvae were fully active in saline for at least 5 h, as there was no significant (*P* > 0.05, repeated measures ANOVA with Bonferroni post test) decline in the proportion of this condition until 6 h of observation (Fig. 1). At 6 h, the proportion of sluggish larvae was significantly increased, with the proportion of paralyzed/dead larvae increasing rapidly by the 7th hour, and by the 9th hour the proportion of paralyzed/dead larvae constituted 90% of the total. Thus, there was excellent longevity of mosquito larvae in this assay, particularly compared to the cut *C. elegans* preparation, which typically lasted about 30 min [5].

Video 1Headless larva.
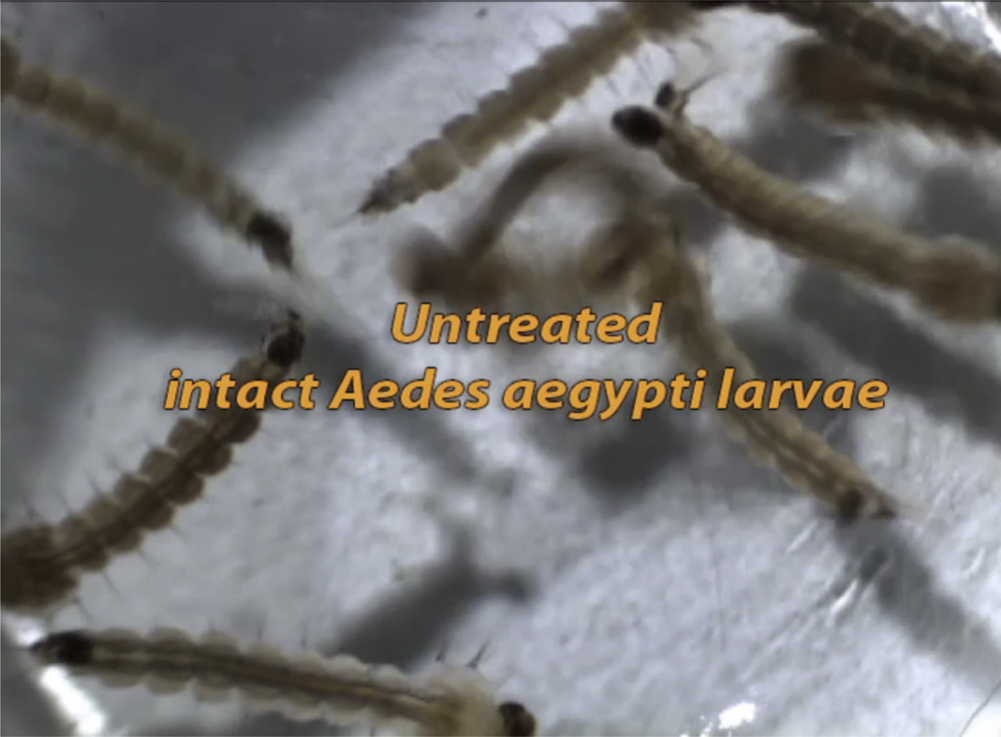

## Validation of the assay: paralytic responses to toxicants

**Fig. 2:**
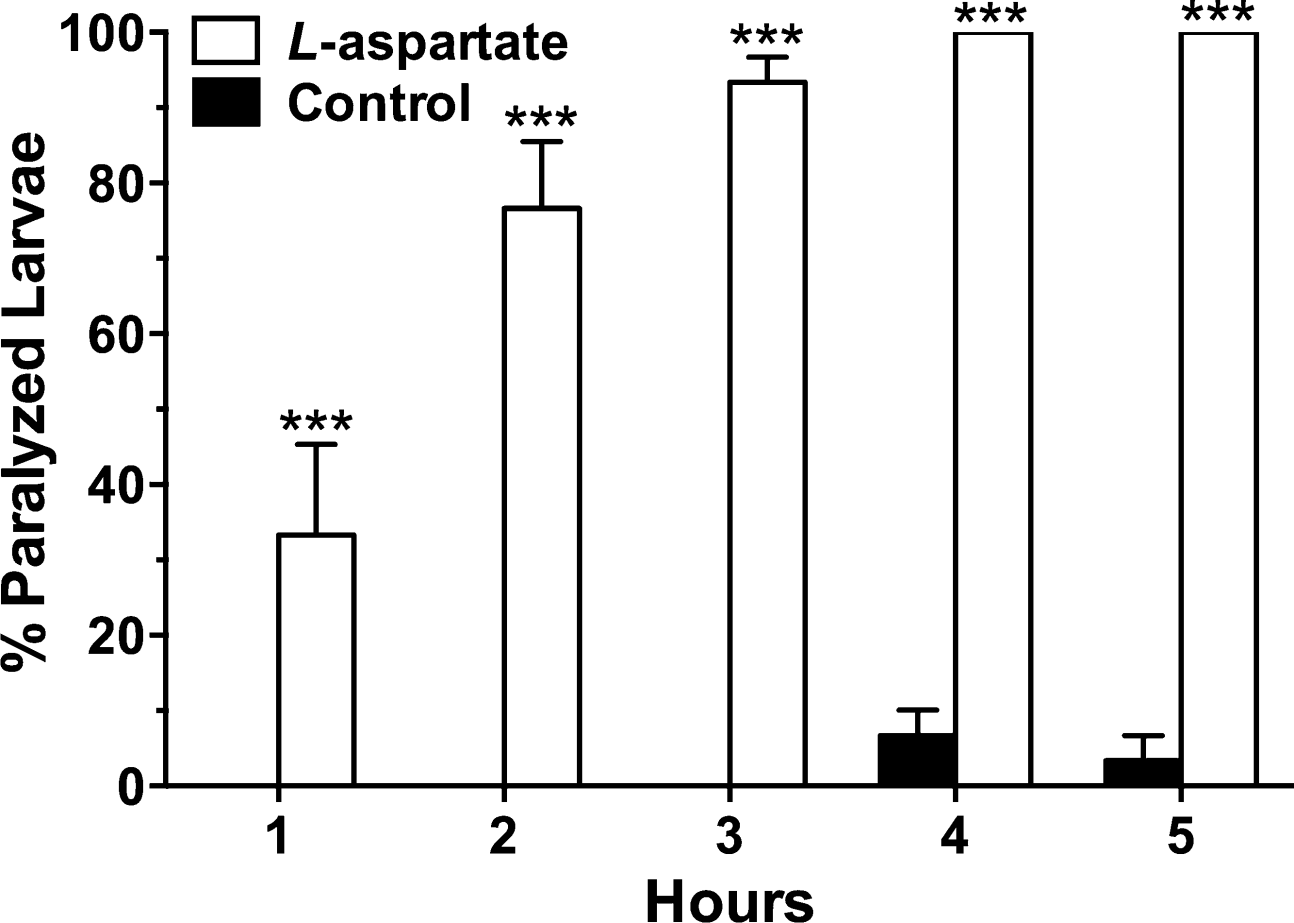
Progressive paralysis of headless *Aedes aegypti* larvae in saline treated with 10 ppm L-aspartate acid for 5 h. Bars are means (*n* = 3 batches of 10 larvae each) with SEM. One way ANOVA with Bonferroni posttest was performed using Prizm software. Asterisks indicate a significant increase (***P < 0.001) in paralysis with pairwise comparisons at each of the indicated time points for larvae exposed to l*-*aspartate, compared to matched controls.

The method distinguished between spastic and flaccid types of paralysis. Larvae that suffered a spastic paralysis assumed a bent or twisted posture, while in flaccid paralysis, the larvae were straight and relaxed. l-aspartate, a water soluble amino acid agonist at dipteran muscle receptors [7] was tested against headless larvae. Stock solutions of the experimental compound were prepared in saline and test concentrations were formulated by adding an appropriate volume of the agonist stock to the saline bath containing the larvae. The motility of larvae was monitored for 5 h, a time period where >90% of untreated headless larvae were still actively motile (Fig. 1), as described above. l-aspartate was a paralytic agent causing flaccid paralysis (Video 1). SAS Probit analysis (SAS 9.3, SAS Institute, Cary, NC, US) was used to determine that the median paralytic concentration (PC_50_) for larvae exposed to l-aspartate was 4.2 with 95% confidence limits of 1.3–13.0 ppm (*χ*^2^ = 5.9; slope = 0.49 + 0.092; df = 6) at the 5 h time point. In separate experiments with 10 ppm l-aspartate, all headless larvae were found paralyzed in 4 h (Fig. 2). In contrast, l-aspartate had no effect on intact larvae at a concentration of 1000 ppm during a 24 h exposure period.

## Validation of the assay: quantification of larval body movement

**Fig. 3:**
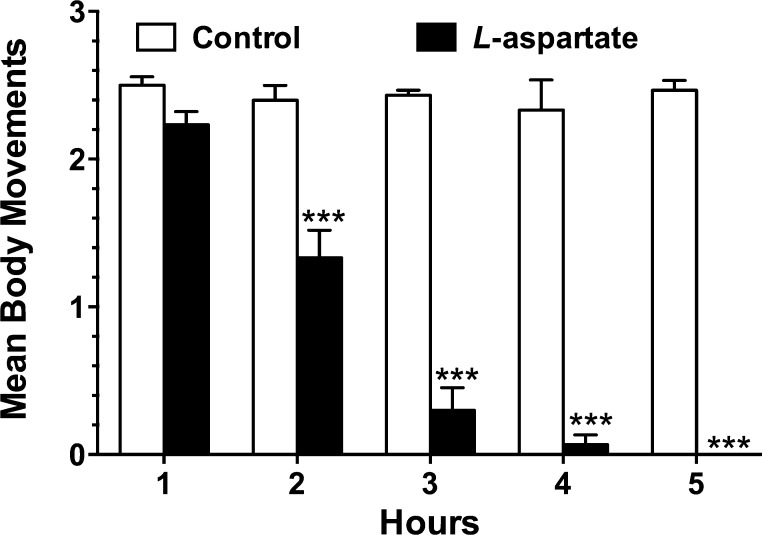
Quantification of larval body movement in headless *Aedes aegypti* larvae treated with 10 ppm l-aspartate for 5 h. Bars are means (*n* = 3 batches of 10 larvae each) with SEM. One way ANOVA with Bonferroni posttest was performed using Prizm software (***P < 0.001), with pairwise comparisons between control and l*-*aspartate treatments at each time point, compared to matched controls.

The assay could also quantify the degree of swimming impairment by counting movements of a larva for 3 s after probing them with an insect pin (Fig. 3). Larvae treated with 10 ppm l-aspartate, if not paralyzed, produced an average of 2.2 movements in the first hour of exposure, but movements gradually diminished to zero at the 5th hour of treatment, consistent with the paralysis results of Fig. 2. In contrast, the untreated larvae preserved motility function and still produced about 2.5 movements within 3 s at the 5th hour (Fig. 3). Exposure of headless mosquito larvae to aspartate reduced larval body movement approximately 2-fold after 2 h, and larval body movement was almost absent after 4 h of exposure. There was no statistically significant difference in the control response over the 5 h time course of the experiment.

This method utilized headless mosquito larvae to study the actions of polar pharmacological compounds. As it is often impossible to assay compounds on intact organisms due to poor cuticular or gut penetration in a feeding assay, the present method provided drug access to internal tissues, along with a convenient way to assess biological activity. This method may also be utilized for *in vivo* pharmacological studies using other small, delicate aquatic insects where an injection method might prove difficult and tedious.
