# Supplementary figures and images for: A method for assessing chemically-induced paralysis in headless mosquito larvae
Source: MethodsX. 2014 Dec 12;2:19–23. doi: 10.1016/j.mex.2014.12.002 (PMC4703516; doi:10.1016/j.mex.2014.12.002)

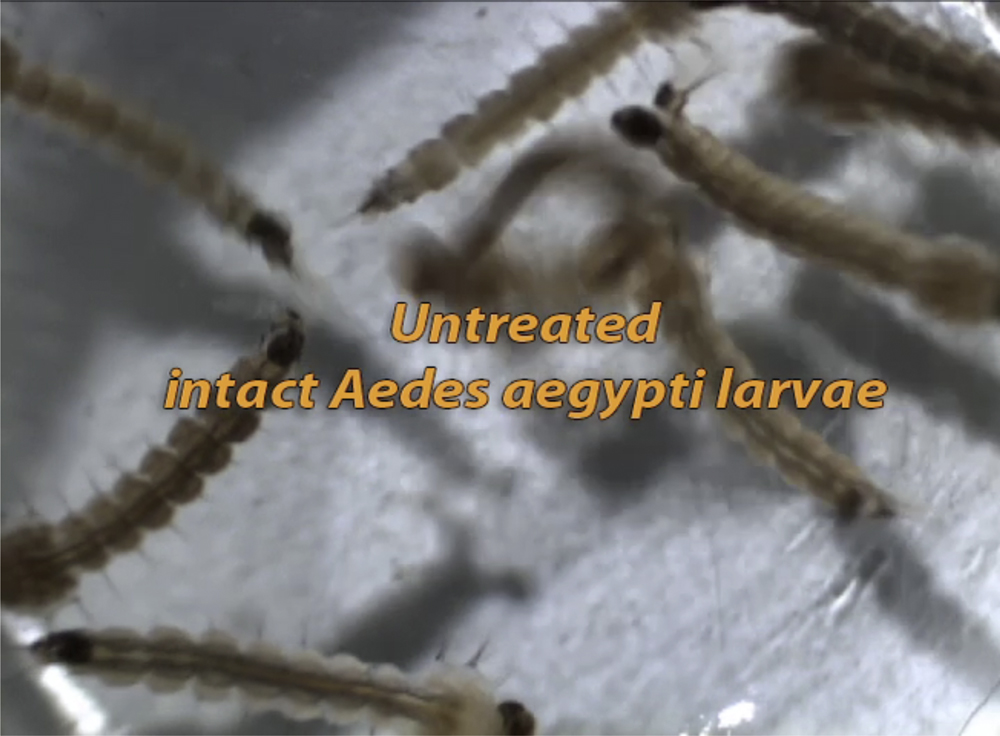

Supplement: Video 1 — Headless larva. [file mmc1.jpg]
